# Vaccination Rates and Influencing Factors in Patients with Axial Spondyloarthritis and Immunosuppressive Treatment—A Survey-Based Cross-Sectional Study

**DOI:** 10.3390/vaccines12070756

**Published:** 2024-07-09

**Authors:** Tuğba Ocak, Selin İldemir Ekizoğlu, Burcu Yağız, Belkıs Nihan Coşkun, Ediz Dalkılıç, Yavuz Pehlivan

**Affiliations:** 1 Department of Rheumatology, Faculty of Medicine, Uludag University, Nilüfer 16285, Bursa, Turkey; burcuyilmaz_84@hotmail.com (B.Y.); belkisnihanseniz@hotmail.com (B.N.C.); edizinci@hotmail.com (E.D.); drypehlivan@gmail.com (Y.P.); 2 Department of Internal Medicine, Faculty of Medicine, Uludag University, Nilüfer 16285, Bursa, Turkey; drselinildemir@gmail.com

**Keywords:** axial spondyloarthritis, immunosuppressive therapy, survey, vaccination

## Abstract

Patients with axial spondyloarthritis (axSpA) who receive immunosuppressive therapy are at risk of infection due to impaired immune function and immunosuppressive medication. Vaccination plays a crucial role in preventing infections in this population. However, vaccination rates and factors influencing vaccination uptake in axSpA patients still need to be adequately studied. This study was designed to determine the vaccination rates of vaccines covered by health insurance in this particular group in Turkey and attitudes towards vaccines and infections. This survey included 199 patients with axSpA who visited our outpatient clinic in June, July, and August 2023 and received biologic and targeted synthetic disease-modifying antirheumatic drugs. The mean age of the participants was 43.7 ± 0.7 years, and the majority were male (66.3%). The majority of the patients were vaccinated against COVID-19 (85.4%), followed by hepatitis B (41.2%), influenza (20.1%), and pneumococcal pneumonia (10.5%). While awareness of COVID-19 vaccination was widespread (100%), knowledge of other vaccines was lower (hepatitis B 80.9%, influenza 70.3%, pneumococcal 60.3%, respectively). Educational interventions targeting patients and healthcare professionals are needed to improve vaccination rates in this population. Our findings emphasize the need for strategies to increase vaccination rates in axSpA patients receiving immunosuppressive therapy. Removing barriers to vaccination and raising awareness of the importance of vaccination are critical to optimizing vaccination practices in this vulnerable population.

## 1. Introduction

Axial spondyloarthritis (axSpA) is a form of chronic inflammatory arthritis mainly affecting the spine and sacroiliac joints [1]. Non-steroidal anti-inflammatory drugs (NSAID) are the first choice in treating axSpA. In NSAID-resistant cases, immunosuppressive therapies such as biologic and targeted synthetic disease-modifying antirheumatic drugs (bDMARDs and tsDMARDs, respectively) are required [2]. These agents significantly improve work productivity and activity limitation in this disease, which occurs in young age groups [3].

Patients with rheumatic and musculoskeletal diseases have a higher risk of infection because their immune function is impaired due to the underlying disease and the immunosuppressive drugs used in treatment [4]. Studies show that the most critical side effect of biological drugs in patients with axSpA is the risk of severe infection [5]. On the other hand, the infection and the necessity to discontinue immunosuppressive treatment during the infection period may exacerbate the disease. Many infections that frequently occur in rheumatologic diseases with complications are among the diseases that can be prevented by vaccination [6]. Vaccines have long been used to reduce illness caused by common viral and bacterial pathogens. Standardized vaccination schedules for children and adults have become established [7,8]. Adults with rheumatologic diseases, especially those receiving immunosuppressive therapy, are a particular group for whom vaccination is indicated [6].

In the coronavirus disease 2019 (COVID-19) pandemic, the importance of vaccination in preventing the spread and reducing the severity of the disease in people with rheumatic diseases has been emphasized [9,10]. The European Alliance of Associations for Rheumatology (EULAR) vaccination recommendations, updated in 2019 and 2021, recommend vaccinations for patients with autoimmune inflammatory rheumatic diseases, including axSpA [9,10]. In the guidelines for the vaccination of adults drawn up by the Turkish Society for Infectious Diseases and Clinical Microbiology, rheumatological diseases have been listed in a separate section since 2016 for axSpA patients receiving immunosuppressive treatment. Among the vaccines recommended by EULAR for autoimmune inflammatory rheumatologic diseases, influenza, pneumococcal pneumonia, hepatitis B, and COVID-19 vaccines are covered by health insurance in our country [11].

Despite the recommendations, vaccination is insufficient in patients with inflammatory rheumatic diseases, including axSpA [12,13]. To our knowledge, there are few studies on vaccination rates and vaccination awareness in axSpA patients receiving immunosuppressive therapy. In some of these studies, the patient population with axSpA receiving biological therapy was small [14,15,16]. Although a survey by Stoffel et al. included many patients, only pneumococcal vaccination rates and predictors were evaluated [17]. Determining vaccination rates and barriers to vaccination in this population receiving immunosuppressive therapy could be essential to improve vaccination. Therefore, in this study, we aimed to investigate the vaccination rates of influenza, pneumococcal pneumonia, hepatitis B, and COVID-19 covered by health insurance in our country, patients’ attitudes towards vaccines and infections, and barriers to vaccination for axSpA patients receiving immunosuppressive therapy at our center.

## 2. Materials and Methods

### 2.1. Study Design and Population

This cross-sectional study was conducted using consecutive sampling of patients employed at the rheumatology outpatient clinic in June, July, and August 2023. Patients over 18 years old were diagnosed with axSpA according to the Assessment of Spondyloarthritis International Society (ASAS) classification included in the study [18]. The exclusion criteria included individuals with Turkish reading and comprehension difficulties, visual or auditory impairments, neurological or mental conditions that hindered comprehension of or response to the questions, or patients who declined to participate in the research. As a result, 199 patients were included in the study. AxSpA patients receiving immunosuppressive therapy are followed up at least every three months in our outpatient clinic. The immunosuppressive medication of the vaccinated patients was suspended for two weeks before vaccination, in accordance with the vaccination recommendations [11]. Before data collection, every participant was provided with information about the research during an individual interview, and a signed agreement for their participation was acquired. Participation was optional, and participants were advised of their prerogative to discontinue their involvement in the trial without impacting their treatment or benefits. The questionnaire was completed face-to-face to assess all patients’ attitudes towards vaccination. The survey was designed and conducted following the recommendations of Gaur et al. [19]. After obtaining a comprehensive medical history, detailed demographic, anthropometric, and clinical data were collected. Laboratory parameters, including human leukocyte antigen (HLA), erythrocyte sedimentation rate (ESR), C-reactive protein (CRP), hemoglobin (Hb), and white blood count (WBC) were examined.

### 2.2. Disease Activity and Functional Assessment

Disease activity was assessed based on a detailed medical history, a physical examination, and laboratory results. The Bath Ankylosing Spondylitis Disease Activity Index (BASDAI) [20], Bath Ankylosing Spondylitis Functional Index (BASFI) [21], and Health Assessment Questionnaire (HAQ) scales [22] were calculated.

### 2.3. Questionnaire

A questionnaire was developed containing 69 questions with predefined answer options. Five rheumatologists reviewed the draft questionnaire to ensure the accuracy of the questions. Ten axSpA patients also reviewed the questionnaire. The questions were revised accordingly. After giving informed consent, patients were asked questions about age, gender, anthropometric measures such as height and weight, smoking and alcohol consumption, education level, marital status, whom they lived with, monthly family income, axSpA diagnosis time, history of immunosuppressive therapy for axSpA and infection history. Furthermore, the questionnaire contained closed questions on knowledge about influenza, pneumococcal, hepatitis B, and COVID-19 vaccines, the source of information, and attitudes toward vaccines and infections. Vaccine hesitancy of the patients was also evaluated with six questions in the questionnaire. The questions for each vaccine were as follows: “Vaccine may cause side effects”; “Vaccine or its adjuvants exacerbate or flare axSpA”; “Vaccine or its adjuvants may reduce the effectiveness of the immunosuppressive treatment”; “Afraid of vaccination”; “Vaccine is not effective for axSpA patients”; and “Vaccine is unnecessary for axSpA patients”. The vaccine-hesitant group included patients who answered “yes” to at least one of these closed-ended yes/no questions.

### 2.4. Statistical Analysis

The statistical analysis used SPSS (Statistical Package for Social Sciences) version 26.0. The normality of variables was assessed using the Kolmogorov–Smirnov test. Quantitative data were expressed as mean ± standard deviation for normal distribution and median (interquartile range, IQR) for non-normal distribution. The Mann–Whitney U and independent sample *t*-test were employed for quantitative variables, while the chi-squared test was used for qualitative variables. The type I error level was adjusted using the Bonferroni correction, with α* = 0.008 for statistical comparisons. Multivariate logistic regression analysis (backward LR) was used for variables with a *p*-value below 0.25 in the univariate analysis. While interpreting the results of the multivariate analysis, a type I error rate of 5% was considered.

## 3. Results

A total of 199 patients, 66.3% (*n* = 132) of whom were male, with a mean age of 43.7 ± 0.7 years and a mean body mass index (BMI) of 26.0 ± 4.6 kg/m^2^, were included in the study. The characteristics of the patients included in the study are listed in Table 1. The median duration of disease was 13.8 (1.8–40) years, and the median duration of immunosuppressive treatment was 9.7 (1.0–22.8) years. The most commonly used immunosuppressive therapy was etanercept 32.7% (*n* = 65), followed by infliximab 24.6% (*n* = 49), golilumab 13.6% (*n* = 27), adalilumab 12.6% (*n* = 25), certolizumab 9% (*n* = 18), secukinumab 5% (*n* = 10), and tofacitinib 2.5% (*n* = 5).

AxSpA was associated with concurrent diseases in 25.1% (*n* = 50) of patients. The most common diseases were hypertension in 13.1% (*n* = 26), diabetes mellitus in 3% (*n* = 6), chronic renal failure, in 2.5% (*n* = 5), familial Mediterranean fever, in 2.5% (*n* = 5), ischemic heart disease, in 2% (*n* = 4), malignancy, in 1.5% (*n* = 3), chronic obstructive pulmonary disease, in 1% (*n* = 2), and heart failure in 0.5% (*n* = 1).

When the vaccination status of the patients was evaluated, the most common receiving vaccine was the COVID-19 vaccine, with a frequency of 85.4% (*n* = 170). The hepatitis B vaccine was the second most common vaccine received, with 41.2% (*n* = 82), followed by the influenza vaccine, with 20.1% (*n* = 40), and the pneumococcal vaccine, with 10.5% (*n* = 21). When the patients’ knowledge of vaccines was evaluated, it was found that all the patients were aware of the COVID-19 vaccine. Of the patients, 80.9% (*n* = 161) knew about the hepatitis B vaccine, 70.3% (*n* = 140) about the influenza vaccine, and 60.3% (*n* = 120) about the pneumococcal vaccine.

### 3.1. Laboratory Features, Disease Activity, and Functional Assessment of the Study Population

The mean values of the BASDAI and BASFI scales evaluating disease activity, HAQ scores assessing physical function, and laboratory results, including ESR, CRP, Hb, and WBC, are shown in Table 2. The analyzed BASDAI was 1.61, the BASFI was 1.43, and the HAQ was 0.09.

### 3.2. Characteristics and Factors Associated with Patients Receiving Influenza Vaccination

The characteristics of the patients with and without the influenza vaccination, knowledge about the influenza vaccination, their sources of information, and their attitudes towards vaccination and influenza infection are shown in Table 3. For those who had received the influenza vaccine, the ages were older (*p* < 0.001). All the people who had received an influenza vaccination were informed about the vaccine. The rate of information about the vaccine from healthcare professionals, television, radio, and newspapers was significantly higher among vaccinated than non-vaccinated people (*p* < 0.001 and *p* < 0.001, respectively). Among the individuals who had received an influenza vaccination, the belief that the vaccine was safe for axSpA patients was significantly higher (*p* < 0.001). Among those who had not been vaccinated against influenza, the belief that the vaccination was unnecessary was significantly higher (*p* < 0.001). The fear of having influenza and the belief that influenza can lead to severe infections was significantly higher in those who had received an influenza vaccination (*p* < 0.001 and *p* < 0.001, respectively).

### 3.3. Characteristics and Factors Associated with Patients Receiving Pneumococcal Vaccination

The characteristics of the patients with and without pneumococcal vaccination, their knowledge about pneumococcal vaccinations, their sources of information, and their attitudes towards vaccination and pneumococcal pneumonia infection are shown in Table 4. The ages of those who had received the pneumococcal vaccine were higher (*p* = 0.001). All the people who had received a pneumococcal vaccine were informed about the vaccine. Those who had received the vaccine were significantly more likely to report having received information about the vaccine from healthcare professionals (*p* < 0.001). Those who had received the pneumococcal vaccine were significantly more likely to believe that the vaccine was safe for patients with axSpA (*p* < 0.001). Those not vaccinated against pneumococcus were significantly more likely to believe the vaccine was unnecessary (*p* < 0.001). The fear of having pneumococcal pneumonia and the belief that pneumococcal pneumonia can lead to severe infections was significantly greater in those who had received pneumococcal vaccination (*p* < 0.001 and *p* < 0.001, respectively).

### 3.4. Characteristics and Factors Associated with Patients Receiving Hepatitis B Vaccination

The characteristics of the patients with and without hepatitis B vaccination, their knowledge about hepatitis B vaccination, their sources of information, and their attitudes towards vaccination and hepatitis B infection are shown in Table 5. All those who had received hepatitis B vaccination were informed about the vaccine. Moreover, the proportion of individuals who had received information about the vaccine from healthcare professionals was significantly higher among those who had been vaccinated than among those who had not (*p* = 0.001). The belief in the safety of the hepatitis B vaccine among the patients with axSpA was significantly higher among those who had received a hepatitis B vaccine (*p* < 0.001). Conversely, among those who had not received the hepatitis B vaccine, the perception that the hepatitis B vaccine reduced the impact of immunosuppressive medications, the fear of vaccination, the belief that the vaccination was not effective, and the belief that the vaccination was unnecessary were significantly higher, *p* < 0.001, *p* = 0.004, and *p* < 0.001, respectively). Additionally, the fear of having hepatitis B and the belief that hepatitis B could lead to severe infections were greater among those who had been vaccinated against hepatitis B (*p* < 0.001 and *p* < 0.001, respectively).

### 3.5. Characteristics and Factors Associated with Patients Receiving COVID-19 Vaccination

The characteristics of the patients with and without COVID-19 vaccination, their knowledge about the COVID-19 vaccine, their sources of information, and their attitudes towards COVID-19 infection and vaccination are shown in Table 6. All the patients had been informed about the COVID-19 vaccine. The belief that the COVID-19 vaccine was safe for axSpA patients was significantly greater among those who had received the vaccine (*p* = 0.003). For those who had not been vaccinated against COVID-19, the possibility of side effects from the vaccine, the fear of vaccination, the belief that the vaccination was not effective, and the belief that the vaccination was unnecessary were significantly greater (*p* < 0.001, *p* < 0.001, *p* = 0.005 and *p* < 0.001 respectively). The fear of being infected with COVID-19 and the belief that infection could lead to severe infections was significantly higher among the vaccinated (*p* < 0.001 and *p* < 0.001, respectively).

### 3.6. Analysis of Factors Influencing Vaccine Hesitancy

General vaccination hesitancy was detected in 19 (9.5%) patients, influenza vaccination hesitancy in 130 (65.3) patients, pneumococcal vaccination hesitancy in 132 (66.3%) patients, hepatitis B vaccination hesitancy in 111 (55.8%) patients, and COVID-19 vaccination hesitancy in 36 (81,9%) patients. The logistic regression analysis results for influenza and COVID-19 vaccination hesitancy are shown in Table 7 and Table 8, respectively. No risk factors were shown for general vaccination, pneumococcal, and hepatitis B vaccination hesitancy in the univariate analysis. Therefore, a multivariate analysis was not performed for these vaccinations. Age of ≥45 years was shown as a risk factor for increasing influenza vaccination hesitancy (odds ratio (OR), 2.627; 95% confidence interval (CI), 1.338–5.158; *p* = 0.005). The presence of comorbidities was shown as a negative risk factor in influenza vaccination hesitancy ((OR), 0.463; 95% (CI), 0.222–0.962; *p* = 0.039). Male gender was found to be borderline statistically significant for COVID-19 vaccination hesitancy in the multivariate analysis (*p* = 0.053).

## 4. Discussion

This is the first study to investigate the vaccination rates of four different vaccines and patient attitudes towards these vaccines and infections in this particular group of patients with axSpA receiving bDMARDs and tsDMARDs, and in such a large number of patients. Firstly, we found that the COVID-19 vaccination rate in this patient group was high, at 85.4% of the patients, which may have been due to increased awareness and attention to COVID-19 vaccination during the ongoing pandemic. Although other vaccines, such as influenza, pneumococcal, and hepatitis B vaccines, are also available and recommended for axSpA patients receiving bDMARDs and tsDMARDs, the lower uptake of these vaccines indicates potential gaps in vaccination practices.

In the COVID-19 group, where vaccine uptake was high, all the patients received information from health professionals. The patients vaccinated with influenza, pneumococcal, and hepatitis B vaccines were also more likely to receive information from healthcare professionals than the patients who had not been vaccinated. In a multicenter observational study on rheumatic diseases, the rate of reluctance to accept the COVID-19 vaccine was 13.6%, while this rate decreased to 8.8% when information was provided by healthcare professionals [23]. Studies by Gaur et al. and Yurttaş et al. highlighted that vaccination rates can increase when healthcare professionals recommend it [24,25]. Increasing the rate of education about vaccination by healthcare professionals is one of the modifiable risk factors. Healthcare professionals do not inform their patients about vaccination due to a lack of education and awareness among healthcare professionals [26,27,28]. The uptake of vaccinations can be improved by better informing and educating healthcare professionals about vaccinations and by informing patients about this topic [16].

In our study, the COVID-19 vaccine was followed by the vaccines against hepatitis B, influenza, and pneumococcus. According to the vaccination recommendations for adults in our country, the recommendation for the hepatitis B vaccine dates back to the past. This vaccination has been provided free of charge since 1998 by the Ministry of Health. The pneumococcal vaccine is the most recent vaccine to have been provided free of charge to immunocompromised patients, since 2016 [11]. The order in which the vaccines were included in the payment scope in our country is similar to the vaccination rates. The reason for this similarity could be that with the inclusion of the vaccine in the payment scope, accessibility to vaccines has improved, and vaccination awareness has increased among health professionals and patients.

In our study, we also identified several factors associated with vaccine uptake and attitudes towards vaccination. Those vaccinated with influenza and pneumococcal vaccines were older in age. Depending on age, many studies have reported higher vaccine uptake and coverage in older people [29,30,31,32,33]. The reasons for this could be that with increasing age, people have more comorbidities, more hospital visits, and more communication with healthcare professionals, and perceive themselves to be at risk of infection [29,33]. In addition, the fact that older people have knowledge and awareness of the vaccination recommendation, the fact that these recommendations do not reach young people, or the fact that young people ignore the recommendations could also play a role [29]. In our country, influenza and pneumococcal vaccinations are recommended for people aged >65 years. They are provided free of charge by the Ministry of Health [11]. Vaccination rates can be increased by strengthening communication with healthcare professionals and improving payment terms for vaccinations.

One of the barriers to vaccination is the patient’s attitude towards vaccination [34]. In our study, those who were vaccinated against influenza, pneumococcal pneumonia, hepatitis B, and COVID-19 believed that the vaccine was safe. Our analysis also uncovered some beliefs and concerns patients held about vaccines. Those who had not been vaccinated against COVID-19 believed the vaccine was ineffective and could cause side effects. In addition, those who had not been vaccinated against, hepatitis B, and COVID-19 feared the vaccine. Addressing these concerns through patient education and open communication with healthcare professionals is essential to promote vaccine acceptance.

In our study, the non-vaccine group for all the evaluated vaccines believed that the vaccine was unnecessary. AxSpA patients receiving bDMARDs and tsDMARDs undergo regular outpatient visits every three months. These visits provide opportunities to inform patients about the necessity and importance of vaccination, potentially increasing vaccine uptake rates.

Vaccine hesitancy is one of the ten threats to global health established by the World Health Organization (WHO) [35]. Vaccine-hesitant individuals may refuse some vaccines or delay vaccination [36]. Our study found the highest vaccine hesitation rate for the pneumococcal vaccine, which also had the lowest vaccine uptake rate. The presence of comorbidities was a negative risk factor for vaccine hesitancy. When age was categorized, ≥45 years of age was shown to be an increased risk factor for vaccine hesitancy. Interestingly, the ages were found to be higher among the influenza vaccine receivers compared to the non-receivers. This may have been because other factors affect vaccination uptake, such as patients’ thoughts and perceptions about influenza infection.

Our results show that there are complicated reasons why axSpA patients receiving immunosuppressive therapy do not receive the vaccinations recommended by the guidelines. In addition to vaccination, patients’ perceptions of the severity of infections may also influence vaccination status [37,38]. The patients vaccinated against influenza, pneumococcal pneumonia, hepatitis B, and COVID-19 were afraid of being infected and feared that infections could lead to severe problems. Vaccination rates can be increased by informing patients about vaccines and the severity of infections.

### Limitations and Strengths

Our study has some limitations. The fact that vaccination status and attitudes are based on self-reporting may have led to recall, reporting, and social desirability biases. In addition, the study’s cross-sectional design and the fact that it was conducted in a single center limit the generalizability of the results to other areas. Zoster vaccination is recommended for all patients undergoing immunomodulant therapy. However, the zoster vaccine was licensed in Turkey in March 2024. Therefore, the zoster vaccine could not be evaluated. The strength of our study is that the questionnaires were completed through face-to-face interviews with patients. Additionally, demographic, clinical, and laboratory characteristics, comorbidities, and disease-activity markers were presented in our study to provide a comprehensive view of the patients.

## 5. Conclusions

There are barriers to optimal vaccine uptake in axSpA patients receiving immunosuppressive therapy. Although COVID-19 vaccination rates are reasonable, vaccination rates for hepatitis B, influenza, and pneumococcal disease are not optimal. Increasing vaccination rates in this patient group by providing information about the efficacy, safety, and necessity of the vaccine by healthcare professionals during rheumatology visits and raising awareness of the vaccine may help prevent vaccine-preventable infections in this immunocompromised group.

## Figures and Tables

**Table 1:** Characteristics of the participants (*n*: 199).

Age, years (mean ± std deviation)	43.7 ± 0.7
Males, *n* (%)	132 (66.3)
BMI (kg/m^2^, mean ± std deviation)	26.0 ± 4.6
Educational level, *n* (%)	
Primary school	26 (13.1)
Middle school	46 (23.1)
High school	76 (38.2)
Collage or above	51 (25.6)
Marial status, *n* (%)	
Unmarried	35 (17.6)
Married	149 (74.9)
Divorced or widowed	15 (7.5)
Family income per month (₺)	
≤15,000	69 (34.7)
15,000–30,000	101 (50.8)
≥30,000	29 (14.5)
Coresident, *n* (%)	
Living with family members, *n* (%)	176 (88.4)
Living with friends	1 (0.5)
Living alone	22 (11.1)
Presence of comorbidities, *n* (%)	50 (25.1)
Smoking *n* (%)	97 (48.7)
Alcohol *n* (%)	31 (15.6)
Emergency-room visits due to infection, *n* (%)	52 (26.1)
Hospitalization due to infection, *n* (%)	10 (5)
Disease duration, years, median (min–max)	13.8 (1.8–40)
Immunosuppressive treatment duration, years, median (min–max)	9.7 (1.0–22.8)
Last immunsupressive treatment, *n* (%)	
Etanercept	65 (32.7)
Infliximab	49 (24.6)
Golilumab	27 (13.6)
Adalilumab	25 (12.6)
Certolizumab	18 (9)
Secukinumab	10 (5)
Tofacitinib	5 (2.5)
NSAID with immunosuppressive treatment	98 (49.2)

Std = standard min (minimum) max (maximum), BMI = body mass index, NSAID = non-steroidal anti-inflammatory drugs.

**Table 2:** Clinical and laboratory parameters of patients with axial spondyloarthritis (*n*: 199).

	Minimum	Maximum	Mean	Standard Deviation
BASDAI	0	6.20	1.61	1.03
BASFI	0	5.30	1.43	0.86
HAQ	0	1.00	0.09	0.17
ESR (mm/h)	2.00	79.00	14.42	13.42
CRP (mg/L)	1.10	126.80	8.96	13.24
Hb (g/dL)	8.20	16.90	13.84	1.76
WBC (10^3^/mL)	4.79	15.30	8.41	1.96

BASDAI = Bath Ankylosing Spondylitis Disease Activity Index, BASFI = Bath Ankylosing Spondylitis Functional Index, HAQ = Health Assessment Questionnaire, ESR = erythrocyte sedimentation rate, CRP = C-reactive protein, Hb = hemoglobin, WBC = white blood count.

**Table 3:** Characteristics and factors associated with patients receiving influenza vaccination (*n*: 199).

	Receiving Influenza Vaccine (*n* = 40)	Not Receiving Influenza Vaccine (*n* = 159)	*p*-Value
Age, years (mean ± std deviation)	49.4 (27.8–73.2)	42.2 (18.7–68.7)	**<0.001**
Gender (female/male)	16/24	51/108	0.343
Comorbidities (present/absent)	15/25	35/124	0.044
Immunsupressive treatment duration, years (mean ± std deviation)	9.8 ± 0.57	9.3 ± 0.34	0.639
Having heard of vaccination *n* (%)	40 (100)	100 (62.9)	**<0.001**
Information source of vaccine *n* (%)			
Health professionals	37 (92.5)	82 (51.6)	**<0.001**
TV, radio or news paper	7 (17.5)	5 (3.1)	**<0.001**
Internet search or social media	13	36	0.196
Family or friends	7	9	0.014
Influenza vaccine is safe for axSpA patients *n* (%)	20 (50)	5 (3.1)	**<0.001**
Influenza vaccine may cause side effects *n* (%)	1 (2.5)	26 (16.4)	0.022
Influenza vaccine or its adjuvants exacerbate or flare axSpA *n* (%)	1 (2.5)	12 (7.5)	0.248
Influenza vaccine or its adjuvants may reduce the effectiveness of the immunosuppressive treatment *n* (%)	1 (2.5)	12 (7.5)	0.248
Afraid of influenza vaccination *n* (%)	0 (0)	9 (5.7)	0.014
Influenza vaccine is not effective for axSpA patients *n* (%)	1 (2.5)	25 (15.7)	0.027
Influenza vaccine is unnecesssary for axSpA patients *n* (%)	1 (2.5)	73 (45.9)	**<0.001**
Have no time for influenza vaccine	1 (2.5)	13 (8.2)	0.210
Afraid of being infected with influenza, *n* (%)	19 (47.5)	6 (3.7)	**<0.001**
Influenza can cause serious problems *n* (%)	20 (50)	12 (7.5)	**<0.001**

AxSpA = axial spondyloarthropathy. Significant results are shown in bold.

**Table 4:** Characteristics and factors associated with patients receiving pneumococcal vaccination (*n*: 199).

	Receiving Pneumococcal Vaccine (*n* = 21)	Not Receiving Pneumococcal Vaccine (*n* = 178)	*p*-Value
Age, years (mean ± std deviation)	50.7(29.8–65.8)	42.8(18.7–73.2)	**0.001**
Gender (female/male)	15/6	52/126	**<0.001**
Comorbidities (present/absent)	9/21	41/137	0.048
Immunsupressive treatment duration, years (mean ± std deviation)	8 ± 0.75	9.7 ± 0.31	0.686
Having heard of vaccination *n* (%)	21 (100)	99 (55.6)	**<0.001**
Information source of vaccine *n* (%)			
Health professionals	20 (95.2)	90 (50.6)	**<0.001**
TV, radio or news paper	3 (14.2)	3 (1.7)	0.017
Internet search or social media	3 (14.2)	24 (13.4)	0.919
Family or friends	3 (14.2)	3 (1.7)	0.017
Pneumococcal vaccine is safe for axSpA patients *n* (%)	12 (57.1)	5 (2.8)	**<0.001**
Pneumococcal vaccine may cause side effects *n* (%)	3 (14.2)	36 (20.2)	0.517
Pneumococcal vaccine or its adjuvants exacerbate or flare axSpA *n* (%)	3 (14.2)	16 (8.9)	0.435
Pneumococcal vaccine or its adjuvants may reduce the effectiveness of the immunosuppressive treatment *n* (%)	4 (19)	13 (7.3)	0.069
Afraid of pneumococcal vaccination *n* (%)	0 (0)	4 (2.2)	0.488
Pneumococcal vaccine is not effective for axSpA patients *n* (%)	4 (19)	17 (9.5)	0.180
Pneumococcal vaccine is unnecesssary for axSpA patients *n* (%)	0	75 (42.1)	**<0.001**
Have no time for pneumococcal vaccine *n* (%)	0	12 (6.7)	0.620
Afraid of being infected with Streptococcus pneumoniae *n* (%)	10 (47.6)	6 (3.4)	**<0.001**
Pneumococcal pneumoniae can cause serious problems *n* (%)	11 (52.3)	9 (5)	**<0.001**

AxSpA = axial spondyloarthropathy. Significant results are shown in bold.

**Table 5:** Characteristics and factors associated with patients receiving hepatitis B vaccination (*n*: 199).

	Receiving Hepatitis B Vaccine (*n* = 82)	Not Receiving Hepatitis B Vaccine (*n* = 117)	*p*-Value
Age, years (mean ± std deviation)	43.3 (20.8–73.2)	43.9 (18.7–68.7)	0.645
Gender (female/male)	32/50	35/82	0.181
Comorbidities (present/absent)	21/61	29/88	0.895
Immunsupressive treatment duration, years (mean ± std deviation)	8.7 ± 0.44	9.8 ± 0.38	0.084
Having heard of vaccination *n* (%)	82 (100)	79 (67.5)	**<0.001**
Information source of vaccine *n* (%)			
Health professionals	81 (98.8)	100 (85.5)	**0.001**
TV, radio or news paper	2 (2.4)	5 (45.4)	0.489
Internet search or social media	28 (34.1)	28 (24)	0.115
Family or friends	5 (6)	7 (5.9)	0.973
Hepatitis B vaccine is safe for axSpA patients *n* (%)	63 (76.8)	3 (2.6)	**<0.001**
Hepatitis B vaccine may cause side effects *n* (%)	1 (1.2)	12 (10.2)	0.011
Hepatitis B vaccine or its adjuvants exacerbate or flare axSpA *n* (%)	3 (3.7)	16 (13.7)	0.018
Hepatitis B vaccine or its adjuvants may reduce the effectiveness of the immunosuppressive treatment *n* (%)	1 (1.2)	19 (16.2)	**<0.001**
Afraid of hepatitis B vaccination *n* (%)	0 (0)	11 (9.4)	**0.004**
Hepatitis B vaccine is not effective for axSpA patients *n* (%)	3 (3.7)	17 (14.5)	0.012
Hepatitis B vaccine is unnecesssary for axSpA patients *n* (%)	0 (0)	55 (47)	**<0.001**
Have no time for Hepatitis B vaccine *n* (%)	0 (0)	2 (1.7)	0.513
Afraid of being infected with hepatitis B *n* (%)	34 (41.5)	2 (1.7)	**<0.001**
Hepatitis B can cause serious problems *n* (%)	67 (81.7)	5 (4.2)	**<0.001**

AxSpA = axial spondyloarthropathy. Significant results are shown in bold.

**Table 6:** Characteristics and factors associated with patients receiving COVID-19 vaccination (*n*: 199).

	Receiving COVID-19 Vaccine (*n* = 170)	Not Receiving COVID-19 Vaccine (*n* = 29)	*p*-Value
Age, year (mean ± std deviation)	43.4 ± 0.8	39.9 ± 1.8	0.691
Gender (female/male)	63/107	4/25	0.015
Comorbidities (present/absent)	45/125	5/24	0.290
Immunsupressive treatment duration, years (mean ± std deviation)	9.7 ± 0.31	8.4 ± 0.79	0.584
Having heard of vaccination *n* (%)	170 (100)	29 (100)	-
Information source of vaccine *n* (%)			
Health professionals	170 (100)	29 (100)	-
TV, radio or news paper	166 (97.6)	27 (93)	0.212
Internet search or social media	112 (65.8)	19 (65.5)	0.969
Family or friends	160 (94.1)	29 (100)	0.363
COVID-19 vaccine is safe for axSpA patients *n* (%)	42 (24.7)	0 (0)	**0.003**
COVID-19 vaccine may cause side effects *n* (%)	7 (4.1)	15 (3.4)	**<0.001**
COVID-19 vaccine or its adjuvants exacerbate or flare axSpA *n* (%)	3 (1.7)	3 (10.3)	0.041
COVID-19 vaccine or its adjuvants may reduce the effectiveness of the immunosuppressive treatment *n* (%)	4 (2.3)	1 (3.4)	0.549
Afraid of COVID-19 vaccination *n* (%)	0 (0)	3 (10.3)	**<0.001**
COVID-19 vaccine is not effective for axSpA patients *n* (%)	2 (1.1)	4 (13.8)	**0.005**
COVID-19 vaccine is unnecesssary for axSpA patients *n* (%)	0 (0)	7 (24.1)	**<0.001**
Have no time COVID-19 vaccine *n* (%)	0	1	0.146
Afraid of being infected with COVID-19	80 (47)	0 (0)	**<0.001**
COVID-19 can cause serious problems	129 (75.8)	1 (3.4)	**<0.001**

AxSpA = Axial spondyloarthropathy Significant results are shown in bold.

**Table 7:** Univariate and multivariate logistic regression analyses of influenza vaccination hesitancy.

Factor		Univariate Analysis Univariate Analysis			Multivariate Analysis Grade 3–4		
		OR	95% CI	*p*	OR	95% CI	*p*
Age (Years)	≥45 (RC) vs. <45	2.009	1.089–3.705	0.026	2.627	1.338–5.158	0.005
Sex	Male (RC) vs. female	1.190	0.645–2.198	0.577			
Educational level	University (RC) vs. non-university	1.083	0.553–2.123	0.816			
Marial status	Unmarried (RC) vs. married	0.824	0.424–1.602	0.568			
Family income per month (₺)	>15,000 (RC) vs. ≤15,000	1.111	0.603–2.045	0.737			
Coresident	Living with members (RC) vs. living alone	0.383	1.124–1.180	0.095	0.350	0.111–1.105	0.073
Presence of comorbidities	Present (RC) vs. absent	0.656	0.340–1.268	0.210	0.463	0.222–0.962	0.039
Smoking	Present (RC) vs. absent	1.045	0.661–1.651	0.851			
Alcohol	Present (RC) vs. absent	0.800	0.458–1.396	0.432			
Disease duration	Years	0.994	0.952–1.039	0.805			
Immunosuppressive treatment duration	Years	0.989	0.922–1.062	0.770			

RC = reference category, OR = odds ratio, CI = confidence interval.

**Table 8:** Univariate and multivariate logistic regression analyses of COVID-19 vaccination hesitancy.

Factor		Univariate Analysis Univariate Analysis			Multivariate Analysis Grade 3–4		
		OR	95% CI	*p*	OR	95% CI	*p*
Age (Years)	≥45 (RC) vs. <45	0.714	0.338–1.508	0.378			
Sex	Male (RC) vs. female	0.495	0.237–1.030	0.060	0.481	0.229–1.011	0.053
Educational level	University (RC) vs. non-university	1.590	0728–3.471	0.245	-	-	-
Marial status	Unmarried (RC) vs. married	0.902	0.431–2.282	0.985			
Family income per month (₺)	>15,000 (RC) vs. ≤15,000	0.926	0.436–1.966	0.841			
Coresident	Living with members (RC) vs. living alone	5.176	0.673–39.803	0.114	5.412	0.698–41.945	0.106
Presence of comorbidities	Present (RC) vs. absent	1.183	0.525–2.664	0.685			
Smoking	Present (RC) vs. absent	0.815	0.458–1.450	0.486			
Alcohol	Present (RC) vs. absent	0.858	0.533–2.128	0.858			
Disease duration	Years	0.964	0.909–1.022	0.217	-	-	-
Immunosuppressive treatment duration	Years	0.986	0.903–1.076	0.750			

RC = reference category, OR = odds ratio, CI = confidence interval.

## Data Availability

The data presented in this study are available on request from the corresponding author.
